# *catena*-Poly[[[tetra­aqua­magnesium]-*trans*-μ-[(piperazine-1,4-diium-1,4-di­yl)bis­(methyl­ene)]di­phospho­nato] hemihydrate]

**DOI:** 10.1107/S1600536813018722

**Published:** 2013-07-13

**Authors:** Lars-Hendrik Schilling, Norbert Stock

**Affiliations:** a Institut für Anorganische Chemie, Christian-Albrechts-Universität Kiel, Max-Eyth-Strasse 2, 24118 Kiel, Germany

## Abstract

The structure of the title polymer, }[Mg(C_6_H_14_N_2_O_6_P_2_)(H_2_O)_4_]·0.5H_2_O}_*n*_, is based on centrosymmetric MgO_6_ octahedra, which are linked by [(piperazine-1,4-diium-1,4-di­yl)bis­(methyl­ene)]di­phospho­nate ligands, forming chains parallel to [1-1-1]. These chains are connected *via* hydrogen bonds primarily formed between the phospho­nate groups and water mol­ecules. The latter constitute four of the corners of the MgO_6_ polyhedra and bind to the O atoms of the phospho­nate groups of neighbouring chains. The lattice water molecule is disordered around an inversion centre, exhibiting an occupancy of 0.25.

## Related literature

For related magnesium structures, see: Wharmby *et al.* (2012 ▶). For related *N*,*N*′-piperaziniumbis(methyl­ene­phospho­nates), see: Choi *et al.* (1994 ▶); Groves *et al.* (2005*a* ▶,*b* ▶); Groves, Stephens *et al.* (2006 ▶); Groves, Miller *et al.* (2006 ▶); LaDuca *et al.* (1996 ▶); Serre *et al.* (2006 ▶); Soghomonian *et al.* (1995 ▶); Wang *et al.* (2004 ▶); Wharmby *et al.* (2012 ▶). As a result of their flexible coordination behaviour, organic linker mol­ecules containing phospho­nate groups allow the synthesis of a multitude of inorganic-organic hybrid materials, see: Gagnon *et al.* (2012 ▶).
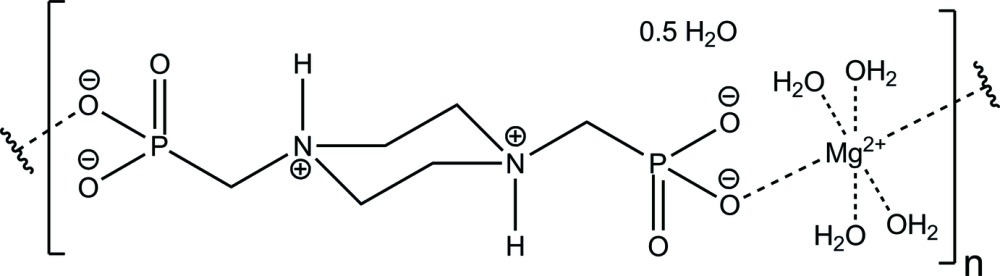


## Experimental

### 

#### Crystal data

[Mg(C_6_H_14_N_2_O_6_P_2_)(H_2_O)_4_]·0.5H_2_O
*M* *_r_* = 377.51
Triclinic, 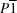

*a* = 6.6296 (5) Å
*b* = 6.8074 (6) Å
*c* = 8.7962 (7) Å
α = 94.579 (6)°
β = 103.326 (6)°
γ = 106.552 (6)°
*V* = 365.75 (5) Å^3^
*Z* = 1
Mo *K*α radiation
μ = 0.40 mm^−1^
*T* = 293 K
0.21 × 0.12 × 0.04 mm

#### Data collection

Stoe IPSD-2 diffractometer
Absorption correction: numerical (*X-SHAPE* and *X-RED32*; Stoe & Cie, 2008 ▶) *T*_min_ = 0.886, *T*_max_ = 0.974
6973 measured reflections
1957 independent reflections
1727 reflections with *I* > 2σ(*I*)
*R*_int_ = 0.036

#### Refinement

*R*[*F* ^2^ > 2σ(*F* ^2^)] = 0.030
*wR*(*F* ^2^) = 0.080
*S* = 1.01
1957 reflections
103 parameters
H-atom parameters constrained
Δρ_max_ = 0.43 e Å^−3^
Δρ_min_ = −0.43 e Å^−3^

### 

Data collection: *X-AREA* (Stoe & Cie, 2008 ▶); cell refinement: *X-AREA*; data reduction: *X-AREA*; program(s) used to solve structure: *SHELXS97* (Sheldrick, 2008 ▶); program(s) used to refine structure: *SHELXL97* (Sheldrick, 2008 ▶); molecular graphics: *XP* in *SHELXTL* (Sheldrick, 2008 ▶) and *DIAMOND* Brandenburg (2011 ▶); software used to prepare material for publication: *XCIF* in *SHELXTL*.

## Supplementary Material

Crystal structure: contains datablock(s) I, global. DOI: 10.1107/S1600536813018722/cq2003sup1.cif

Structure factors: contains datablock(s) I. DOI: 10.1107/S1600536813018722/cq2003Isup2.hkl

Additional supplementary materials:  crystallographic information; 3D view; checkCIF report

## Figures and Tables

**Table 1:** Hydrogen-bond geometry (Å, °)

*D*—H⋯*A*	*D*—H	H⋯*A*	*D*⋯*A*	*D*—H⋯*A*
N1—H1*N*1⋯O3^i^	0.86	1.84	2.6187 (16)	150
O4—H1*O*4⋯O3^ii^	0.82	1.99	2.7956 (15)	166
O4—H2*O*4⋯O2	0.82	1.88	2.6733 (17)	164
O5—H1*O*5⋯O2^iii^	0.82	1.89	2.7032 (17)	172
O5—H2*O*5⋯O2^iv^	0.82	1.99	2.7667 (18)	158

Symmetry codes: (i) 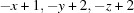; (ii) 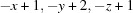; (iii) 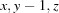; (iv) 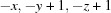.

**Fig. 1.**
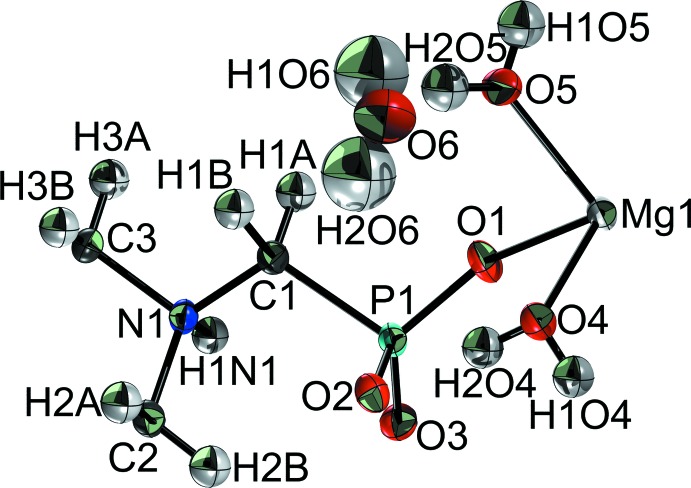
: Asymmetric unit of the title compound with labeling and displacement ellipsoids drawn at the 50% probability level.

**Fig. 2.**
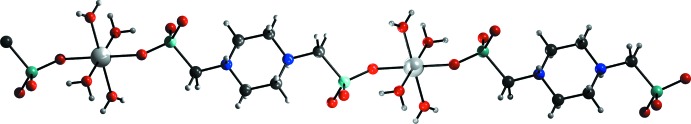
: Chain-formed building unit of [Mg(H2O)4(H2L)] . 0.5 H2O.

## supplementary crystallographic information

### Comment

Due to their flexible coordination behaviour, organic linker molecules containing phosphonate groups allow the synthesis of a multitude of inorganic-organic hybrid materials (Gagnon *et al.*, 2012). In this context, the ligand *N,N'*-piperazinebis(methylenephosphonic acid) (H_2_O_3_P—CH_2_—NC_4_H_8_N—CH_2_—PO_3_H_2_ = H_4_L) has been the subject of intense interest, since its use has led to a number of dense metal phosphonates (Groves, Stephens *et al.*, 2006, Groves *et al.*, 2005*a*, 2005*b*, Choi *et al.*, 1994, LaDuca *et al.*, 1996, Soghomonian *et al.*, 1995, Wang *et al.*, 2004) as well as porous ones (Groves, Miller *et al.*, 2006, Serre *et al.*, 2006). The compounds [Co_2_(H_2_O)_2_L] ^.^ 5.1 H_2_O (denoted Co-STA-12) and [Mg_2_(H_2_O)_2_L] ^.^ 3.97 H_2_O (denoted CAU-2) are highly porous with micropore volumes of 0.14 cm^3^ g^-1^ and 0.20 cm^3^ g^-1^, respectively (Wharmby *et al.*, 2012). Investigation of the system MgCl_2_^.^ 6 H_2_O / H_4_L / base / H_2_O led to the formation of CAU-2. We have now been able to isolate a new compound, [Mg(H_2_O)_4_(H_2_L)] ^.^ 0.5 H_2_O, in this system, which is obtained from slightly acidic reaction mixtures (4 ≤ pH ≤ 6.5). Optimization of the reaction conditions (concentration and reaction time) led to the formation of single crystals. The asymmetric unit of the crystal structure is depicted in Fig. 1.

[Mg(H_2_O)_4_(H_2_L)] ^.^ 0.5 H_2_O adopts a one-dimensional structure containing alternating inorganic and organic building units of MgO_6_ polyhedra and *N,N'*-piperaziniumbis(methylenephosphonate) ions (Fig. 2). The Mg^2+^ ions are octahedrally coordinated by six oxygen atoms (O1, O4, O5 and their symmetry equivalents), of which four (O4 and O5) belong to water molecules while two (O1) belong to phosphonate groups of the ligand molecules. The charge of the structure is balanced by protons connected to the N atoms of the ligand making it a quaternary amine. In addition, a further water molecule is found on a partially occupied position.

### Experimental

A reaction mixture of MgCl_2_^.^ 6 H_2_O (13.55 mg, 50 µmol), *N,N'*-piperazinebis(methylenephosphonic acid) (36.55 mg, 100 nmol), potassium hydroxide (16.83 mg, 300 nmol) and 1.5 ml water was placed in a 2 ml Teflon-lined autoclave. Subsequently the reactor was heated from room temperature to 130 °C (heating rate 1 °C min^-1^), the temperature was held for 52 h and then slowly lowered to room temperature over a period of 12 h. The resulting colourless crystals were collected by filtration and analysed *via* single-crystal XRD. Yield: 32%.

### Refinement

All H atoms of C—H groups were located in difference maps but were positioned with idealized geometry and were refined isotropically with *U*_iso_(H) = 1.2 *U*_eq_(C) using a riding model with C—H = 0.97 Å for aliphatic H atoms. The water H atoms were located in difference maps, their bond lengths were set to ideal values of 0.82 Å and they were refined using a riding model with *U*_iso_(H) = 1.5 *U*_eq_(O). The N—H H atom was located in a difference map but was positioned with idealized geometry and was refined isotropic with *U*_iso_(H) = 1.2 *U*_eq_(C) using a riding model with N—H = 0.86 Å.

### Crystal data

**Table:**

[Mg(C_6_H_14_N_2_O_6_P_2_)(H_2_O)_4_]·0.5H_2_O	*Z* = 1
*M**_r_* = 377.51	*F*(000) = 199
Triclinic, *P*1	*D*_x_ = 1.714 Mg m^−^^3^
Hall symbol: -P 1	Mo *K*α radiation, λ = 0.71073 Å
*a* = 6.6296 (5) Å	Cell parameters from 1270 reflections
*b* = 6.8074 (6) Å	θ = 1.2–29.8°
*c* = 8.7962 (7) Å	µ = 0.40 mm^−^^1^
α = 94.579 (6)°	*T* = 293 K
β = 103.326 (6)°	Needle, colorless
γ = 106.552 (6)°	0.21 × 0.12 × 0.04 mm
*V* = 365.75 (5) Å^3^	

### Data collection

**Table:**

Stoe IPSD-2 diffractometer	1957 independent reflections
Radiation source: fine-focus sealed tube	1727 reflections with *I* > 2σ(*I*)
Graphite monochromator	*R*_int_ = 0.036
ω scan	θ_max_ = 29.2°, θ_min_ = 3.2°
Absorption correction: numerical (*X-SHAPE* and *X-RED32*; Stoe & Cie, 2008)	*h* = −9→9
*T*_min_ = 0.886, *T*_max_ = 0.974	*k* = −9→9
6973 measured reflections	*l* = −12→12

### Refinement

**Table:**

Refinement on *F*^2^	Primary atom site location: structure-invariant direct methods
Least-squares matrix: full	Secondary atom site location: difference Fourier map
*R*[*F*^2^ > 2σ(*F*^2^)] = 0.030	Hydrogen site location: inferred from neighbouring sites
*wR*(*F*^2^) = 0.080	H-atom parameters constrained
*S* = 1.01	*w* = 1/[σ^2^(*F*_o_^2^) + (0.0352*P*)^2^ + 0.2734*P*] where *P* = (*F*_o_^2^ + 2*F*_c_^2^)/3
1957 reflections	(Δ/σ)_max_ < 0.001
103 parameters	Δρ_max_ = 0.43 e Å^−^^3^
0 restraints	Δρ_min_ = −0.43 e Å^−^^3^

### Special details

**Table:**

Geometry. All e.s.d.'s (except the e.s.d. in the dihedral angle between two l.s. planes) are estimated using the full covariance matrix. The cell e.s.d.'s are taken into account individually in the estimation of e.s.d.'s in distances, angles and torsion angles; correlations between e.s.d.'s in cell parameters are only used when they are defined by crystal symmetry. An approximate (isotropic) treatment of cell e.s.d.'s is used for estimating e.s.d.'s involving l.s. planes.
Refinement. Refinement of *F*^2^ against ALL reflections. The weighted *R*-factor *wR* and goodness of fit *S* are based on *F*^2^, conventional *R*-factors *R* are based on *F*, with *F* set to zero for negative *F*^2^. The threshold expression of *F*^2^ > σ(*F*^2^) is used only for calculating *R*-factors(gt) *etc*. and is not relevant to the choice of reflections for refinement. *R*-factors based on *F*^2^ are statistically about twice as large as those based on *F*, and *R*- factors based on ALL data will be even larger.

### Fractional atomic coordinates and isotropic or equivalent isotropic displacement parameters (Å^2^)

**Table:**

	*x*	*y*	*z*	*U*_iso_*/*U*_eq_	Occ. (<1)
Mg1	0.5000	0.5000	0.5000	0.01868 (16)	
P1	0.36611 (6)	0.83891 (6)	0.71790 (4)	0.01741 (10)	
O1	0.47100 (19)	0.67534 (18)	0.69031 (14)	0.0256 (2)	
O2	0.22643 (18)	0.87610 (17)	0.56605 (13)	0.0228 (2)	
O3	0.52336 (14)	1.04094 (13)	0.81824 (11)	0.0225 (2)	
C1	0.17809 (14)	0.73201 (13)	0.83666 (11)	0.0218 (3)	
H1A	0.2264	0.6279	0.8904	0.026*	
H1B	0.0379	0.6240	0.8034	0.026*	
N1	0.1348 (2)	0.89184 (19)	0.94153 (15)	0.0181 (2)	
H1N1	0.2589	0.9558	1.0077	0.022*	
C2	0.0614 (3)	1.0529 (2)	0.85892 (19)	0.0235 (3)	
H2A	−0.0754	0.9870	0.7791	0.028*	
H2B	0.1690	1.1225	0.8065	0.028*	
C3	−0.0313 (3)	0.7900 (2)	1.02369 (19)	0.0218 (3)	
H3A	0.0147	0.6857	1.0786	0.026*	
H3B	−0.1695	0.7211	0.9461	0.026*	
O4	0.30950 (18)	0.64149 (17)	0.34463 (13)	0.0236 (2)	
H1O4	0.3778	0.7291	0.3008	0.028*	
H2O4	0.2647	0.7153	0.3980	0.028*	
O5	0.22487 (19)	0.26098 (18)	0.51161 (16)	0.0309 (3)	
H1O5	0.2383	0.1488	0.5310	0.037*	
H2O5	0.0957	0.2540	0.4922	0.037*	
O6	0.5116 (13)	0.5606 (12)	1.0711 (9)	0.0620 (19)	0.25
H1O6	0.5284	0.5120	1.1538	0.093*	0.25
H2O6	0.5205	0.6822	1.0956	0.093*	0.25

### Atomic displacement parameters (Å^2^)

**Table:**

	*U*^11^	*U*^22^	*U*^33^	*U*^12^	*U*^13^	*U*^23^
Mg1	0.0172 (3)	0.0181 (3)	0.0219 (3)	0.0073 (3)	0.0059 (3)	0.0011 (3)
P1	0.01580 (17)	0.01772 (18)	0.01999 (19)	0.00653 (13)	0.00637 (13)	0.00069 (13)
O1	0.0300 (6)	0.0285 (6)	0.0246 (6)	0.0176 (5)	0.0094 (5)	0.0020 (4)
O2	0.0203 (5)	0.0246 (5)	0.0244 (5)	0.0097 (4)	0.0047 (4)	0.0025 (4)
O3	0.0200 (5)	0.0213 (5)	0.0237 (5)	0.0037 (4)	0.0053 (4)	0.0010 (4)
C1	0.0228 (7)	0.0170 (6)	0.0276 (8)	0.0055 (5)	0.0121 (6)	0.0011 (6)
N1	0.0151 (5)	0.0194 (6)	0.0206 (6)	0.0052 (4)	0.0070 (5)	0.0019 (5)
C2	0.0269 (7)	0.0258 (7)	0.0246 (7)	0.0132 (6)	0.0127 (6)	0.0077 (6)
C3	0.0216 (7)	0.0205 (7)	0.0271 (7)	0.0066 (6)	0.0129 (6)	0.0060 (6)
O4	0.0245 (5)	0.0233 (5)	0.0252 (6)	0.0101 (4)	0.0075 (4)	0.0032 (4)
O5	0.0186 (5)	0.0240 (6)	0.0501 (8)	0.0059 (4)	0.0086 (5)	0.0106 (5)
O6	0.065 (5)	0.059 (4)	0.068 (5)	0.025 (4)	0.019 (4)	0.020 (4)

### Geometric parameters (Å, º)

**Table:**

Mg1—O1^i^	2.0552 (11)	N1—H1N1	0.8600
Mg1—O1	2.0553 (11)	C2—C3^ii^	1.513 (2)
Mg1—O5	2.0991 (12)	C2—H2A	0.9700
Mg1—O5^i^	2.0992 (12)	C2—H2B	0.9700
Mg1—O4^i^	2.1209 (11)	C3—C2^ii^	1.513 (2)
Mg1—O4	2.1209 (11)	C3—H3A	0.9700
P1—O1	1.5027 (11)	C3—H3B	0.9700
P1—O2	1.5242 (12)	O4—H1O4	0.8199
P1—O3	1.5240 (10)	O4—H2O4	0.8201
P1—C1	1.8383	O5—H1O5	0.8200
C1—N1	1.5022 (15)	O5—H2O5	0.8200
C1—H1A	0.9700	O6—O6^iii^	1.395 (16)
C1—H1B	0.9700	O6—H1O6	0.8201
N1—C3	1.4925 (18)	O6—H2O6	0.8200
N1—C2	1.495 (2)		
			
O1^i^—Mg1—O1	180.00 (6)	C3—N1—C2	108.49 (11)
O1^i^—Mg1—O5	90.35 (5)	C3—N1—C1	110.46 (10)
O1—Mg1—O5	89.65 (5)	C2—N1—C1	114.75 (11)
O1^i^—Mg1—O5^i^	89.65 (5)	C3—N1—H1N1	111.5
O1—Mg1—O5^i^	90.35 (5)	C2—N1—H1N1	106.5
O5—Mg1—O5^i^	180.00 (7)	C1—N1—H1N1	105.1
O1^i^—Mg1—O4^i^	89.80 (4)	N1—C2—C3^ii^	110.27 (12)
O1—Mg1—O4^i^	90.20 (4)	N1—C2—H2A	109.6
O5—Mg1—O4^i^	87.19 (5)	C3^ii^—C2—H2A	109.6
O5^i^—Mg1—O4^i^	92.81 (5)	N1—C2—H2B	109.6
O1^i^—Mg1—O4	90.20 (4)	C3^ii^—C2—H2B	109.6
O1—Mg1—O4	89.80 (4)	H2A—C2—H2B	108.1
O5—Mg1—O4	92.81 (5)	N1—C3—C2^ii^	111.06 (12)
O5^i^—Mg1—O4	87.19 (5)	N1—C3—H3A	109.4
O4^i^—Mg1—O4	180.0	C2^ii^—C3—H3A	109.4
O1—P1—O2	113.06 (7)	N1—C3—H3B	109.4
O1—P1—O3	114.25 (6)	C2^ii^—C3—H3B	109.4
O2—P1—O3	112.00 (6)	H3A—C3—H3B	108.0
O1—P1—C1	105.10 (6)	Mg1—O4—H1O4	115.4
O2—P1—C1	106.31 (6)	Mg1—O4—H2O4	108.1
O3—P1—C1	105.20 (5)	H1O4—O4—H2O4	99.6
P1—O1—Mg1	137.40 (7)	Mg1—O5—H1O5	119.2
N1—C1—P1	114.69 (7)	Mg1—O5—H2O5	130.9
N1—C1—H1A	114.7	H1O5—O5—H2O5	109.7
P1—C1—H1A	108.7	O6^iii^—O6—H1O6	119.9
N1—C1—H1B	102.6	O6^iii^—O6—H2O6	133.7
P1—C1—H1B	128.8	H1O6—O6—H2O6	106.3
H1A—C1—H1B	83.9		

Symmetry codes: (i) −*x*+1, −*y*+1, −*z*+1; (ii) −*x*, −*y*+2, −*z*+2; (iii) −*x*+1, −*y*+1, −*z*+2.

### Hydrogen-bond geometry (Å, º)

**Table:**

*D*—H···*A*	*D*—H	H···*A*	*D*···*A*	*D*—H···*A*
N1—H1*N*1···O3^iv^	0.86	1.84	2.6187 (16)	150
O4—H1*O*4···O3^v^	0.82	1.99	2.7956 (15)	166
O4—H2*O*4···O2	0.82	1.88	2.6733 (17)	164
O5—H1*O*5···O2^vi^	0.82	1.89	2.7032 (17)	172
O5—H2*O*5···O2^vii^	0.82	1.99	2.7667 (18)	158
C1—H1*B*···O4^vii^	0.97	2.48	3.4279 (15)	166
C2—H2*B*···O3	0.97	2.55	3.187 (2)	124

Symmetry codes: (iv) −*x*+1, −*y*+2, −*z*+2; (v) −*x*+1, −*y*+2, −*z*+1; (vi) *x*, *y*−1, *z*; (vii) −*x*, −*y*+1, −*z*+1.
